# 2621. Social Risk Factors for RSV-related Hospitalizations in Adults ≥ 50 years of age

**DOI:** 10.1093/ofid/ofad500.2234

**Published:** 2023-11-27

**Authors:** Khalel De Castro, Ashley Tippett, Laila Hussaini, Luis W Salazar, Olivia Reese, Caroline R Ciric, Wensheng Li, Hui-Mien Hsiao, Kathy Stephens, Theda Gibson, Elizabeth Begier, Qing Liu, Robin Hubler, Bradford D Gessner, Benjamin Lopman, Nadine Rouphael, Satoshi Kamidani, Evan J Anderson, Christina A Rostad

**Affiliations:** Emory University School of Medicine, Atlanta, Georgia; Emory University, Atlanta, Georgia; Emory Univeristy, Atlanta, Georgia; Emory University, Atlanta, Georgia; Emory University, Atlanta, Georgia; Emory University, Atlanta, Georgia; Emory University School of Medicine, Atlanta, Georgia; Emory University School of Medicine, Atlanta, Georgia; Emory University School of Medicine, Atlanta, Georgia; Emory University School of Medicine, Atlanta, Georgia; Pfizer Vaccines, Dublin, Dublin, Ireland; Pfizer Inc., Collegeville, Pennsylvania; Pfizer Inc., Collegeville, Pennsylvania; Pfizer Biopharma Group, Collegeville, Pennsylvania; Rollins School of Public Health | Emory University, Atlanta, Georgia; Emory University School of Medicine, Atlanta, Georgia; Emory University School of Medicine and Children's Healthcare of Atlanta, Atlanta, Georgia; Moderna, Inc., Atlanta, Georgia; Emory University School of Medicine and Children's Healthcare of Atlanta, Atlanta, Georgia

## Abstract

**Background:**

Although respiratory syncytial virus (RSV) is a common pathogen in older adults, little is known about the social risk factors for RSV hospitalization in this population. In this study, we sought to evaluate the social determinants of RSV-related hospitalizations in older adults.

**Methods:**

From October 2018 to March 2020, we enrolled patients ≥50 years of age who were admitted with an acute respiratory infection (ARI) or CHF/COPD exacerbation at two Emory University hospitals. Enrolled patients were interviewed regarding their medical and social history and their medical charts were abstracted. Nasopharyngeal and oropharyngeal swabs and standard-of-care specimens were obtained for BioFire® Respiratory Panel analysis. Demographic, interview responses, and selected comorbidities were compared with bivariate analysis and generated a stepwise logistic regression model with inclusion in the model set at 0.05. Statistical analysis was performed using SAS v.9.4.

**Results:**

Of the 1429 enrolled participants, 78 (5.5%) were RSV-positive (Table 1). Compared to RSV-negative participants, those with RSV were more commonly female (66.7% vs. 55.3%, P=0.05), immunocompromised (43.6% vs. 31.5%, P=0.03) and particularly with HIV/AIDS (11.5% vs. 3.5%, P=0.003), and had traveled > 100 miles in the prior 2 weeks (12.8% vs. 6.7%, P=0.04). No significant differences were found between the groups by baseline health status or other comorbidities. Participants with RSV had higher frequency of low to moderate activity at baseline than those who were RSV-negative. No significant differences were identified for those living with children or performing childcare ≥ 6 hours a week. Adjusting for sex, activity frequency, travel, and immunocompromised status, those who were male (OR 1.9, 95%CI 1.16, 3.15), exercised 2-3 times per week (OR 2.3, 95%CI 1.26, 4.38), traveled (OR 2.3, 95%CI 1.09, 4.70), or were immunocompromised (OR 1.8, 95%CI 1.10, 2.85) had greater odds of RSV positivity compared to the reference groups (Table 2).

**Table 1.**
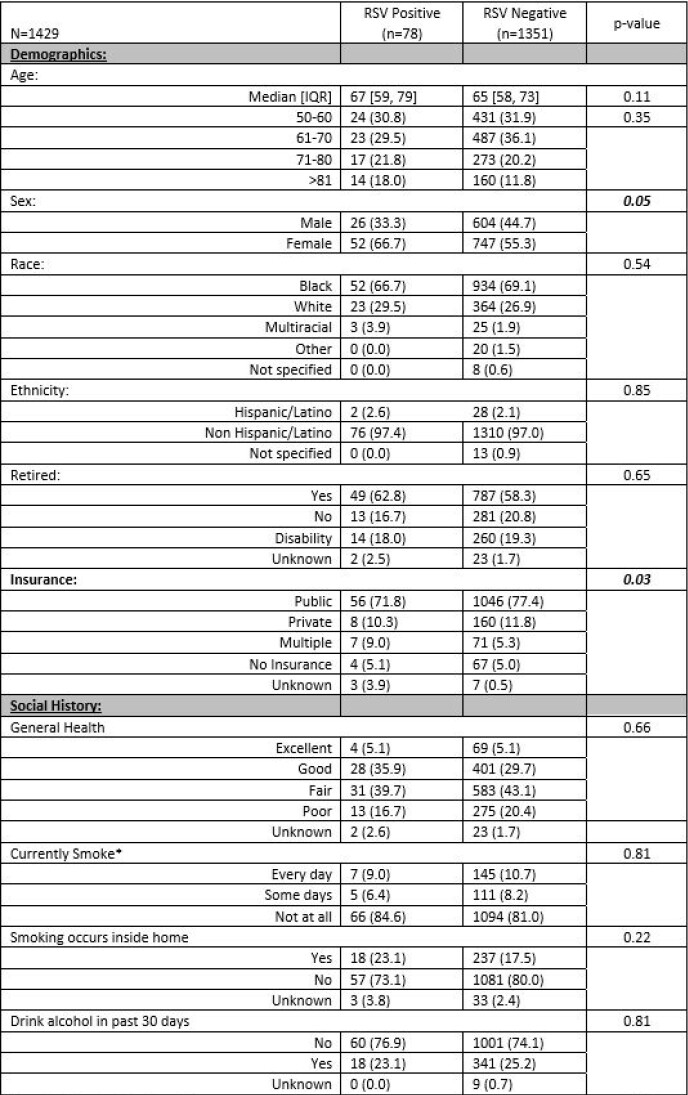
Demographics, comorbidities, and outcomes of older adults (50+yo) admitted to hospital with ARI or CHF/COPD exacerbation by RSV status. *1 missing response, RSV Negative **Immunocompromised defined as patients having: cancer, HIV/AIDS, solid organ transplant, stem cell/bone marrow transplant, long-term steroid use, or other immune decreasing conditions

**Figure:**
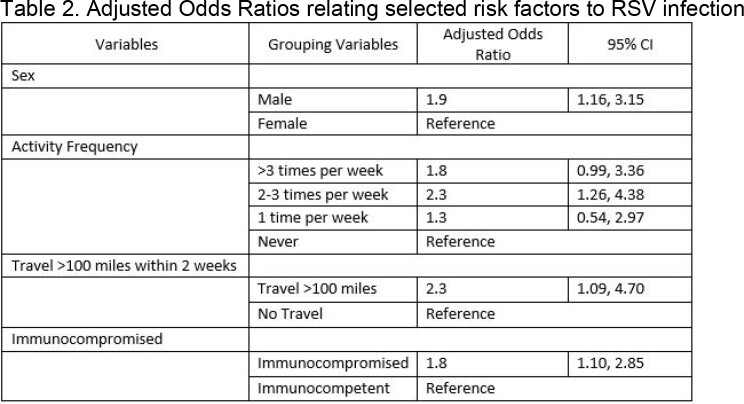

**Conclusion:**

Among ARI hospitalizations, RSV prevalence was higher for female sex, immunocompromised status, and those that travelled within the preceding two weeks. Understanding risk factors for severe RSV hospitalization may inform prevention recommendations.

**Disclosures:**

**Elizabeth Begier, M.D., M.P.H.**, Pfizer: EB is an employee of Pfizer, the sponsor of this study|Pfizer: Stocks/Bonds **Qing Liu, M.S.**, Pfizer Inc.: Stocks/Bonds **Robin Hubler, MS**, Pfizer, Inc.: Employee|Pfizer, Inc.: Stocks/Bonds **Bradford D. Gessner, M.D., M.P.H.**, Pfizer: I am an employee of Pfizer|Pfizer: Stocks/Bonds **Benjamin Lopman, PhD**, Epidemiological Research and Methods, LLC: Advisor/Consultant|Hillevax, Inc: Advisor/Consultant **Nadine Rouphael, MD**, Icon, EMMES, Sanofi, Seqirus, Moderna: Advisor/Consultant **Satoshi Kamidani, MD**, CDC: Grant/Research Support|Emergent BioSolutions: Grant/Research Support|NIH: Grant/Research Support|Pfizer Inc: Grant/Research Support **Evan J. Anderson, MD**, GSK: Advisor/Consultant|GSK: Grant/Research Support|Janssen: Advisor/Consultant|Janssen: Grant/Research Support|Kentucky Bioprocessing, Inc.: Safety Monitoring Board|Moderna: Advisor/Consultant|Moderna: Grant/Research Support|Moderna: Currently an employee|Moderna: Stocks/Bonds|Pfizer: Advisor/Consultant|Pfizer: Grant/Research Support|Sanofi Pasteur: Advisor/Consultant|Sanofi Pasteur: Grant/Research Support|Sanofi Pasteur: Safety Monitoring Board|WCG/ACI Clinical: Data Adjudication Board **Christina A. Rostad, MD**, BioFire Inc.: Grant/Research Support|GlaxoSmithKline Biologicals: Grant/Research Support|Janssen: Grant/Research Support|MedImmune LLC: Grant/Research Support|Meissa Vaccines, Inc.: RSV vaccine technology|Merck & Co., Inc.: Grant/Research Support|Micron Technology, Inc.: Grant/Research Support|Moderna, Inc.: Grant/Research Support|Novavax: Grant/Research Support|PaxVax: Grant/Research Support|Pfizer, Inc.: Grant/Research Support|Regeneron: Grant/Research Support|Sanofi Pasteur: Grant/Research Support

